# Short- and long-term effects of musculoskeletal health education: evidence from the performing arts students

**DOI:** 10.3389/fspor.2025.1637680

**Published:** 2025-07-31

**Authors:** Štefan Adamčák, Michal Marko, Pavol Bartík, Zora Kľocová Adamčáková

**Affiliations:** ^1^ Faculty of Sports Science and Health, Matej Bel University in Banská Bystrica, Banská Bystrica, Slovakia; ^2^ Faculty of Performing Arts, Academy of Arts in Banská Bystrica, Banská Bystrica, Slovakia; ^3^ Regional Public Health Authority in Banská Bystrica, Banská Bystrica, Slovakia

**Keywords:** core stability, health literacy, playing-related musculoskeletal disorders, postural awareness, preventive education, spinal mobility

## Abstract

**Background:**

Performing arts students (i.e., musicians), face elevated risks of performance-related musculoskeletal disorders due to prolonged exposure to static postures, repetitive movements, and lack of ergonomic education. While the benefits of musculoskeletal health education are established in sports, its application and long-term effects in performing arts education remain underexplored. This study aimed to evaluate both short- and long-term effects of musculoskeletal health education on dynamic spine function among female music students.

**Methods:**

Forty-four female music students from the Academy of Arts in Banská Bystrica participated in a non-randomized controlled study. Participants were divided into an experimental group (*n* = 28), which received 8-week musculoskeletal health education (2x/week/45 min), and a control group (*n* = 16), which received no intervention. The education included theoretical and practical components focused on postural awareness and dynamic spine function. Spine mobility was assessed using standardized methods common in medical and physical therapy practice at three time points: pre-test, post-test, and follow-up. Statistical analysis was performed using non-parametric Wilcoxon and Mann–Whitney tests, with significance set at *p* < .05.

**Results:**

The experimental group showed statistically significant improvements in all dynamic spine function tests post-intervention (e.g., Schober's: from 2.71 ± .81 cm to 5.60 ± .68 cm, *p* < .05) with partial retention at follow-up (4.03 ± .79 cm). Significant gains were also observed in Thomayer's test (from −7.46 ± 4.66 cm to −.78 ± 1.39 cm), indicating enhanced spine mobility. In contrast, the control group demonstrated significant declines across all parameters over time. Intergroup comparisons post-intervention and at follow-up revealed significantly better outcomes in the experimental group across all measures (*p* < .05), confirming the effectiveness of education.

**Conclusions:**

Musculoskeletal health education program led to significant short- and long-term effects on dynamic spine function in female music students. The results underscore the importance of integrating such programs into performing arts curricula to prevent musculoskeletal decline and promote health literacy. This study provides compelling evidence that musculoskeletal education, when embedded into arts training, is both a preventive and rehabilitative tool essential for sustaining the physical well-being of performing arts students.

## 1 Introduction

Performing arts students, including musicians (in our case), represent an exceptional and (often) an at-risk population in discussions of occupational health. Such individuals face extraordinary physical and psychological demands during their training and careers (early), often without the comprehensive musculoskeletal health education given to their counterparts in sports (1–3); therefore, performing arts students are affected by musculoskeletal disorders, many of which result from avoidable factors such as poor posture, overuse, and inadequate conditioning (1, 4, 5). Prevalence of performance-related musculoskeletal disorders (PRMDs) in this population, ranging from 60% to 90% depending on instruments, has become subjects of growing concern in performing arts medicine (4, 6). Performing arts students, in particular, musicians, are exposed to prolonged hours of repetitive movements, static loading, and awkward postures without ergonomic adjustments (5, 7, 8). The absence of adequate knowledge results in delayed diagnoses, incorrect management, and acceptance of pain and injury as normal. Challenges like these are intensified by prevailing cultural mindsets within the performing arts that emphasize endurance and performance over individual health (9).

Education is an elementary (i.e., fundamental) pillar in injury prevention (1–3). Musculoskeletal health education encompasses learning about the structure and function of body's muscles, bones, and joints, as well as understanding how to prevent and manage musculoskeletal disorders (5, 10, 11). Research suggests that when implemented early and constantly, musculoskeletal health education can reduce the incidence of PRMDs, improve postural awareness, and enhance endurance level (9, 12). An example is the PRESTO trial, large-scale study evaluating the biopsychosocial prevention program in performing arts students. The intervention, which combined physical training, health education, and behavioral strategy, demonstrated improvements (*p* < .01) in levels of pain and performance-related behaviors (4). Research conducted during an intensive summer music festival showed that brief 90-minute musculoskeletal education program (MEP) led to 32% reduction in pain incidence in the intervention group, while the controls experienced an increase of 8% (*p* < .01) (5). Such results underscore the effectiveness of such programs in high-demand performance settings. In addition to structured programs, educational formats have also proven effective. The El-Poems study from Iran found that an e-learning module focusing on postural behaviors significantly (*p* < .001) decreased musculoskeletal pain and improved ergonomic awareness in performing arts students (9). Including musculoskeletal health education in performing arts training is not just helpful, it is essential, both from teaching and healthcare prospectives. Education that combines different methods, like online learning, hands-on workshops, and movement re-education, is the most effective at encouraging lasting changes in behaviors (8, 13–15).

Musculoskeletal health education programs exist; however, few have examined both the short- and long-term effects on the musculoskeletal system, including dynamic spine function (7, 9, 12, 16–19). Most existing research focuses on short-term results and often lacks follow-up evaluations (4, 20–22). This study seeks to address these gaps by evaluating the short- and long-term effects of musculoskeletal health education on dynamic spine function. By focusing on performing arts students, it contributes to holistic understanding of how such education can foster sustainable health practices within this population.

## 2 Materials and methods

### 2.1 Participants

Considering the probability of participant dropout in longitudinal study (23), the target (i.e., final) population consisted of 44 female music students (100%) enrolled in the bachelor's degree (1st–3rd year) in the Performing Arts (study program) at the Faculty of Performing Arts, Academy of Arts in Banská Bystrica, Slovakia.

Musculoskeletal health education was implemented over a 8-week period, in particular, 2x/week/45 min (Mondays and Fridays). The intervention focused on enhancing postural awareness, with emphasis on dynamic spine function, in 28 students (63.63%) assigned to the experimental group (mean age: 21.23 ± 1.47 years; weight: 55.85 ± 3.57 kg; height: 167.82 ± 2.43 cm). The remaining 16 students (36.37%) were allocated to the control group, which did not receive musculoskeletal health education (mean age: 21.81 ± 1.63 years; weight: 53.81 ± 3.74 kg; height: 169.43 ± 1.78 cm). The target population (*n* = 44, 100%) was recruited using convenience sampling, targeting female students enrolled in the elective course—“*Prevention of Musculoskeletal System 1–2”.* Additional recruitment was carried out through institutional email invitations (24). All 44 students formed homogeneous groups (i.e., experimental and control) in terms of anthropometric data and academic characteristics, ensuring consistency across the sample (Table 1). Convenience sampling enabled recruitment while confirming that all participants (*n* = 44; 100%) met the criteria necessary to address the research questions. All participants had been free of musculoskeletal disorders (i.e., PRMDs) for three months (at least) in advance of study.

**Table 1:** Demographic characteristics of participants (*n* = 44, 100%).

Characteristics	Experimental group	Control group
Anthropometric		
Age (years; *M* *±* *SD*)	21.23 ± 1.47	21.81 ± 1.63
Weight (kg; *M* *±* *SD*)	55.85 ± 3.57	53.81 ± 3.74
Height (cm; *M* *±* *SD*)	167.82 ± 2.43	169.43 ± 1.78
Instruments		
Wind (*N; %*)	12; 42.86%	10; 62.50%
String (*N; %*)	6; 21.42%	2; 12.50%
Keyboard (*N; %*)	10; 35.72%	4; 25%
Practice		
Day (hours; *M* *±* *SD*)	3.21 ± .45	3.43 ± .38
Career (years; *M* *±* *SD*)	14.59 ± 1.26	13.97 ± 1.67

M, mean; SD, standard deviation; N, number.

Ethical approval was granted by Ethics Committee of Artistic and Pedagogical Council of Faculty of Performing Arts, Academy of Arts in Banská Bystrica (Approval No. 001, FMU-AU/24), was carried out under the standards set by the Declaration of Helsinki (25, 26). Written informed consent for participation in this study was obtained from all participants.

### 2.2 Assessments and procedures

The musculoskeletal education program was delivered over a period of 10 weeks, from October 1 to November 24, 2024. Sessions took place 2x/week, in particular, on Tuesdays and Thursdays, and each lasted 45 min. Every session was divided into two parts, in particular, 20 min theoretical component that covered opening concepts, followed by 25 min practical component (5 min warm-up, 15 min main block, 5 min cool-down) focused on applying these concepts through guided exercises and demonstrations. The program functioned as the experimental stimulus for the experimental group, which included 28 students (63.63%). The program was tailored to target specific, predefined musculoskeletal states (S), in particular, dynamic spine function, offering structured approaches to improve female students understanding and competency in this area. In contrast, the control group was composed of 16 students (36.37%) who did not receive any form of intervention. They continued with their regular schedule and academic activities without exposure to the musculoskeletal educational program. This group served as the baseline for comparisons, helping to isolate and evaluate the specific effects of the musculoskeletal education program. Both groups were assessed at three distinct time points to capture changes over time and determine the short- and long-term effects of musculoskeletal health education:

1. Before attending the musculoskeletal education program (pre-test, October 1, 2024).
2. After attending the musculoskeletal education program (post-test, November 24, 2024).
3. 24 weeks after attending the musculoskeletal education program (follow-up, May 8, 2025).

A standardized assessment for evaluating the dynamic spine function was administered at three time points: before (pre-test, October 1, 2024) and after the musculoskeletal education program (post-test, November 24, 2024), and at a 24-week follow-up (May 8, 2025). The assessment evaluates mobility of spine and alignment during movement. It is a widely used method in medical and physical therapy practice and was therefore conducted by Doctor of Medicine (one of the authors). For most tests, the starting position is an upright back posture; if a different position is used, it is specified in the testing:

1. Schober's test (Sch, Lumbar spine)—The distance measured indicates the development and mobility of the lumbar spine. Starting from the L5 vertebra, a point is measured 10 cm cranially in adults and 5 cm cranially in children; both points can be marked on the skin using a dermograph. After the initial marking, the subject bends forward. In individuals with a healthy spine, the distance between the two marked points should increase to 14 cm in adults and 7.5 cm in children. Some authors suggest an alternative method, where the measurement begins at the S1 vertebra, with an extended cranial distance ranging from 10 to 15 cm. If the elongation is less than the expected norm, it may indicate limited mobility or stiffness in the lumbar spine.
2. Stibor's test (St, Lumbar & thoracic spine)—The distance measured indicates the development and mobility of the lumbar and thoracic spine. From the spine of the L5 vertebra (or S1), the distance is measured up to the spine of the C7 vertebra. Both points can be marked with a dermograph. After the measurement, the subject leans forward in a relaxed manner. In a healthy spine, the distance between the two points should increase by 7 to 10 cm. If the elongation is less than the expected norm, it may indicate limited mobility or stiffness in the thoracic and/or lumbar spine.
3. Otto's test (Ot, Thoracic spine)—The Otto's inclination distance assesses the mobility of the thoracic spine during forward bending. The starting point is the spine of the C7 vertebra, from which a point is marked 30 cm caudally using a dermograph. During forward flexion, the distance between the two points should increase by at least 3.5 cm. Otto's recline distance evaluates thoracic spine mobility during backward leaning. The second point is again located 30 cm caudally from the C7 vertebra, and during extension, the distance should decrease by 2.5 cm. The sum of both Otto's distances represents the sagittal mobility index of the thoracic spine. If the sum of the Otto's distances is less than the expected norm, it may indicate reduced sagittal mobility of the thoracic spine, suggesting stiffness or functional limitation in spinal flexion and extension.
4. Thomayer's test (Th, Mobility of spine)—The Thomayer's distance, also known as the forward bend test, provides a general and non-specific assessment of overall spinal mobility. The test is performed from a standing position, where the subject bends forward and the distance from the tip of the third finger to the mat is measured at the maximum point of flexion. During the test, attention must be paid to possible compensatory movements, such as bending at the hips, and/or limitations caused by shortened knee flexors, which may result in the patient bending the knees and experiencing discomfort in the popliteal fossa. In addition to detecting hypomobility, the test may also reveal significant hypermobility, indicated when the subject is able to touch the mat with the entire palm or even the forearm. Such findings suggest a considerable ligamentous disorder. Increased ligament laxity and positive hypermobility findings are more frequently observed in women. A normal result is indicated when the fingers touch the mat; a distance of up to 10 cm is still considered within normal limits. A distance greater than 30 cm is regarded as a clear pathological finding.
5. Lateroflexion (Lat, Lumbar spine)—The bowing test serves as an indicative assessment, providing information about the symmetry and extent of lateral flexion. The subject stands with their back against a wall, arms resting alongside the body with palms facing inward. The subject then performs a lateral bend, and the point reached by the longest finger on each side is marked. This allows for comparison of symmetry and the range of motion in side bending. During side bending, the distance reached by the longest finger from the starting point typically falls between 20 and 22 cm. If the elongation is either less than or greater than the normal range, it may indicate reduced or excessive lateral spinal mobility, or asymmetry between the left and right sides (27).

All sessions for the experimental group were conducted under the direct supervision of the authors. During these sessions, participants received detailed instruction not only on how to perform the practical elements but also on the principles and objectives underlying each component of the program (Table 2). The decision to deliver the program in a group setting was intentional. It was chosen for its cost-effectiveness, its ability to foster peer support and collaboration, and its potential to enhance student engagement and motivation throughout the learning process (28, 29). Musculoskeletal education program was designed to maximize benefits while minimizing injury risks (7).

**Table 2:** Musculoskeletal education program.

Week	Theory	Practice
1	Introduction to anatomy of spine; importance of neutral spine position	**Warm-up:** Gentle spinal rolls and seated breathing **Main block:** Mirror-based posture drills, core activation (abdominal bracing, diaphragmatic breathing) **Cool-down:** Supine spinal decompression, mindful breathing
2	Common postural deviations; ergonomic risk factors during sitting	**Warm-up:** Standing spinal flexion/extension **Main block:** Wall alignment drills, sit-to-stand posture practice, ergonomic posture simulation **Cool-down:** Neck and shoulder release, seated stretches
3	Role of mobility in musculoskeletal (postural) health and movement	**Warm-up:** Cat-cow and thoracic rolls **Main block:** Segmental bridging, thoracic spine rotations, student's pose reach **Cool-down:** Supine twist, diaphragmatic breathwork
4	Lumbopelvic stability and deep core synergy	**Warm-up:** Pelvic tilts and breath-to-core connection **Main block:** Dead bug, bird-dog, front and side planks with cues **Cool-down:** Knees-to-chest stretch, prone relaxation
5	Maintaining posture during movement: walking, lifting	**Warm-up:** Marching in place with spinal awareness **Main block:** Gait retraining, controlled lunges, lifting technique **Cool-down:** Standing spinal elongation, calf/hip flexor stretch
6	Ergonomics and postural habits in academic settings	**Warm-up:** Seated spinal mobilization **Main block:** Desk setup simulations, thoracic extension, scapular retraction exercises **Cool-down:** Wrist, neck, and upper back stretches
7	Identifying and correcting dysfunctional patterns	**Warm-up:** Dynamic trunk rotations **Main block:** Resistance band drills (scapular retraction, spinal extension), posture correction flow **Cool-down:** Side-lying spinal twist, foam rolling
8	Integration of knowledge and long-term posture maintenance	**Warm-up:** Full-body dynamic warm-up **Main block:** Integrated movement flow (mobility + stability), peer posture feedback **Cool-down:** Relaxation in supported supine position, final breathing practice

### 2.3 Data analysis

Evidence from all 44 female students (100%) in the performing arts was organized and presented in structured database formats. Because of small number of participants, the authors used non-parametric tests for comparisons between and within the groups to understand how important the difference was. As the Kolmogorov–Smirnov test evidenced that variables (most) did not have normal distributions, Wilcoxon Test was used for intragroup comparisons between pre-test, post-test and follow-up. Mann–Whitney Test was used for intergroup comparisons between the experimental group (*n* = 28, 63.63%) and the control group (*n* = 16, 36.37%). The *p* value was set at.05 and the effect size (r) was calculated using Wilcoxon Test and Mann–Whitney Test, which is the z value, divided by the total number of observations (30). Descriptive data were reported as group mean values *±* standard deviations (SD). Statistical analysis was carried out using IBM SPSS Version 27 (31).

## 3 Results

Table 3 illustrates the scores of dynamic spine function (*M* *±* *SD*) and intragroup comparisons (*Wilcoxon Test*) before, after, and following the musculoskeletal health education. The units of measurement for all indicators presented in Tables 3, 4 are in centimeters (cm). This is consistent with the standardized methods of assessing spinal mobility used in the study, such as Schober's, Stibor's, Otto's, and Thomayer's tests, as well as left and right lateroflexion. In the experimental group, all assessed parameters of dynamic spine function showed statistically significant improvements following the musculoskeletal education program, with meaningful effect sizes across the Schober's, Stibor's, Otto's, and Thomayer's tests as well as left and right lateroflexion. Schober's test mean improved from 2.71 ± .81 cm at baseline to 5.60 ± .68 cm post-intervention, and maintained a partial gain at follow-up (4.03 ± .79 cm), with significant changes across all time points (*p* < .05). Similar trends were observed in the Stibor's test (before: 6.60 ± .68 cm; after: 9.39 ± .73 cm; follow-up: 7.82 ± .61 cm), indicating enhanced flexibility and spinal mobility. Thomayer's test reflected substantial functional gain in spine mobility, with a marked improvement from −7.46 ± 4.66 cm at baseline to −.78 ± 1.39 cm post-intervention, then regressing slightly to −3.53 ± −3.52 cm at follow-up. Regarding the presence of negative values, in particular, in Thomayer's test, these reflect specific characteristics of the measurement protocol. In this test, the distance from the third fingertip to the floor is recorded. A negative value indicates that the fingers did not reach the floor and represents the shortfall in centimeters. Conversely, a value of zero or a positive number would suggest that the subject touched the floor or extended beyond it, signifying greater spinal flexion or hypermobility. The comparison of negative and positive values in this context is meaningful as it quantifies degrees of mobility and allows tracking of functional improvement. In statistical analyses, these values were handled as continuous variables and interpreted in terms of absolute improvement, regardless of sign, consistent with previous musculoskeletal research standards. Improvements in both left and right lateroflexion, from 19.25 cm (left) and 19.28 cm (right) cm at baseline to 21.78 cm post-intervention, remained relatively stable at follow-up (21.32 cm; left and right). In contrast, the control group demonstrated statistically significant but clinically unfavorable trends, with declines across all measures over time, including in the Schober's test (from 3.31 ± 0.60 to 2.62 ± 0.61 cm) and Thomayer's test (from −10.12 ± 3.13 to −10.62 ± 3.11 cm), suggesting a deterioration in spinal function in the absence of intervention.

**Table 3:** Scores of dynamic spine function (*M* *±* *SD*) and intragroup comparisons (*Wilcoxon test*) before, after, and following musculoskeletal health education.

Experimental group					
Dynamic spine function	Before (*cm*)	After (*cm*)	Follow-up (*cm*)	Wilcoxon (*p*)	Effect size (*r*)
Schober's test (*M* *±* *SD*)	2.71 ± .81	5.60 ± .68	4.03 ± .79	<.05^a^^,^^b^^,^^c^	.62^d^;.62^e^;.61^f^
Stibor's test (*M* *±* *SD*)	6.60 ± .68	9.39 ± .73	7.82 ± .61	<.05^a^^,^^b^^,^^c^	.63^d^;.62^e^;.62^f^
Otto's test (*M* *±* *SD*)	4.39 ± .78	5.85 ± .35	4.75 ± .75	<.05^a^^,^^b^^,^^c^	.62^d^;.56^e^;.27^f^
Thomayer's test (*M* *±* *SD*)	−7.46 ± 4.66	−.78 ± 1.39	−3.53 ± −3.52	<.05^a^^,^^b^^,^^c^	.59^d^;.51^e^;.58^f^
Lateroflexion (*L; M* *±* *SD*)	19.25 ± 1.04	21.78 ± .49	21.32 ± .67	<.05^a^^,^^b^^,^^c^	.59^d^;.42^e^;.59^f^
Lateroflexion (*R; M* *±* *SD*)	19.28 ± .97	21.78 ± .49	21.32 ± .67	<.05^a^^,^^b^^,^^c^	.60^d^;.44^e^;.58^f^
Control group					
Parameters	Before (*cm*)	After (*cm*)	Follow-up (*cm*)	Wilcoxon (*p*)	Effect size (*r*)
Schober's test (*M* *±* *SD*)	3.31 ± .60	3.06 ± .57	2.62 ± .61	<.05^b^^,^^c^	.28^d^;.41^e^;.58^f^
Stibor's test (*M* *±* *SD*)	6.43 ± .81	6.12 ± .71	5.93 ± .77	<.05^a^^,^^c^	.39^d^;.41^e^;.50^f^
Otto's test (*M* *±* *SD*)	3.87 ± 1.02	3.62 ± 1.02	3.60 ± 1.42	<.05	.27^d^;.12^e^;.10^f^
Thomayer's test (*M* *±* *SD*)	−10.12 ± 3.13	−9.62 ± 3.11	−10.62 ± 3.11	<.05^b^	.18^d^;.42^e^;.12^f^
Lateroflexion (*L; M* *±* *SD*)	19.25 ± .85	18.93 ± 1.02	18.62 ± 1.02	<.05^a^^,^^c^	.39^d^;.24^e^;.44^f^
Lateroflexion (*R; M* *±* *SD*)	19.43 ± 1.03	18.87 ± 2.66	18.31 ± 1.66	<.05^b^^,^^c^	.33^d^;.41^e^;.54^f^

^a^ Significant differences (<.05) between pre- and post-test.

^b^ Significant differences (<.05) between post-test and follow-up.

^c^ Significant differences (<.05) between pre-test and follow-up.

^d^ Relationships between pre- and post-test.

^e^ Relationships between post-test and follow-up.

^f^ Relationships between pre-test and follow-up.

L, left; R, right; M, mean; SD, standard deviation; cm, centimeter.

**Table 4:** Scores of dynamic spine function (M ± SD) and intergroup comparisons (Mann–Whitney test) before, after, and following musculoskeletal health education.

Before (Pre-test)				
Dynamic spine function	Experimental (*cm*)	Control (*cm*)	Mann–Whitney (*p*)	Effect size (*r*)
Schober's test (*M* *±* *SD*)	2.71 ± .81	3.31 ± .60	<.05	−.36
Stibor's test (*M* *±* *SD*)	6.60 ± .68	6.43 ± .81	>.05	−.06
Otto's test (*M* *±* *SD*)	4.39 ± .78	3.87 ± 1.02	>.05	−.24
Thomayer's test (*M* *±* *SD*)	−7.46 ± 4.66	−10.12 ± 3.13	<.05	−.35
Lateroflexion (*L; M* *±* *SD*)	19.25 ± 1.04	19.25 ± .85	>.05	−.06
Lateroflexion (*R; M* *±* *SD*)	19.28 ± .97	19.43 ± 1.03	>.05	−.08
After (Post-test)				
Dynamic spine function	Experimental (*cm*)	Control (*cm*)	Mann–Whitney (*p*)	Effect size (*r*)
Schober's test (*M* *±* *SD*)	5.60 ± .68	3.06 ± .57	<.05	−.85
Stibor's test (*M* *±* *SD*)	9.39 ± .73	6.12 ± .71	<.05	−.84
Otto's test (*M* *±* *SD*)	5.85 ± .35	3.62 ± 1.02	<.05	−.87
Thomayer's test (*M* *±* *SD*)	−.78 ± 1.39	−9.62 ± 3.11	<.05	−.85
Lateroflexion (*L; M* *±* *SD*)	21.78 ± .49	18.93 ± 1.02	<.05	−.88
Lateroflexion (*R; M* *±* *SD*)	21.78 ± .49	18.87 ± 2.66	<.05	−.82
Follow-up				
Dynamic spine function	Experimental (*cm*)	Control (*cm*)	Mann–Whitney (*p*)	Effect size (*r*)
Schober's test (*M* *±* *SD*)	4.03 ± .79	2.62 ± .61	<.05	−.69
Stibor's test (*M* *±* *SD*)	7.82 ± .61	5.93 ± .77	<.05	−.81
Otto's test (*M* *±* *SD*)	4.75 ± .75	3.62 ± 1.02	<.05	−.54
Thomayer's test (*M* *±* *SD*)	−3.53 ± −3.52	−10.62 ± 3.11	<.05	−.70
Lateroflexion (*L; M* *±* *SD*)	21.32 ± .67	18.62 ± 1.02	<.05	−.82
Lateroflexion (*R; M* *±* *SD*)	21.32 ± .67	18.31 ± 1.66	<.05	−.80

L, left; R, right; M, mean; SD, standard deviation; cm, centimeter.

Table 4 illustrates the scores of dynamic spine function (*M* *±* *SD*) and intergroup comparisons (*Mann–Whitney Test*) before, after, and following the musculoskeletal health education. Intergroup comparisons between the experimental and control groups at all three assessment points revealed significant differences favoring the intervention. While baseline values were mostly similar, with no significant differences in most parameters except Schober's and Thomayer's tests, the post-intervention measurements showed consistently superior outcomes in the experimental group. For example, in the post-test, the experimental group scored 5.60 ± .68 cm on the Schober's test, in contrast to the control group's 3.06 ± .57 cm (*p* < .05, *r* = −.85), indicating enhanced lumbar flexibility. Comparable improvements were noted in Stibor's and Otto's tests, with effect sizes around −.84 and −.87. Thomayer's test revealed dramatic improvement in forward flexion (−.78 ± 1.39 cm vs. −9.62 ± 3.11 cm; *p* < .05), emphasizing increased reach and spinal mobility. At the 24-week follow-up, although some regression was observed in the experimental group, all measures remained significantly better than those in the control group. Lateroflexion scores in the experimental group were consistently higher than controls (21.32 ± .67 cm vs. 18.62 ± 1.02 cm—left, *p* < .05; 21.32 ± .67 cm vs. 18.31 ± 1.66 cm—right, *p* < .05), reinforcing the sustained benefit of the musculoskeletal health education on lateral spinal mobility.

It is acknowledged that statistically significant differences were observed between the experimental and control groups in two parameters, Schober's and Thomayer's tests, at baseline (*p* < .05). These differences are methodological limitations inherent to the quasi-experimental design and the use of convenience sampling. However, the differences were not clinically substantial and remained within the range of typical biological variation, as reflected in the standard deviations and moderate effect sizes (Schober's: *r* = −.36; Thomayer's: *r* = −.35).

The feasibility of conducting random assignments or exact pre-matching of participants was limited by demographic constraints. At the bachelor's level of Performing Arts education at the Academy of Arts in Banská Bystrica, only 64 female students are enrolled across all three study years. The entire Faculty of Performing Arts includes just 167 students, of whom 93 are women. Consequently, it was not possible to selectively construct two fully homogeneous groups without compromising either the ecological validity of the research or its statistical power. Furthermore, there are only two universities in Slovakia offering specialized higher education in performing arts. This structural limitation significantly narrows the potential research population for studies of this nature. Given this context, the intervention was implemented transparently as a pragmatic evaluation of an educational program under real-world institutional conditions.

Moreover, the primary objective of this quasi-experimental study was to evaluate both short- and long-term effects of musculoskeletal health education in a real-world educational setting. Given this context, the intervention proceeded with full transparency, and baseline disparities were statistically controlled through intragroup (Wilcoxon) and intergroup (Mann–Whitney) analyses. This approach ensured that observed post-intervention changes were not artifacts of initial group differences but genuine outcomes of the educational intervention.

## 4 Discussion

The findings of our study demonstrate an evident, significant (*p* < .05) improvements in dynamic spine function among 28 female music students (63.63%) who participated in musculoskeletal education program. Across all evaluated tests, including Schober's, Stibor's, Otto's, and Thomayer's as well as lateroflexion, the experimental group showed marked progress from baseline to post-intervention, and retained substantial gains at the 24-week follow-up. The results are statistically robust and carry considerable clinical relevance, reflecting functional enhancements in dynamic spine function and posture, both of which are important for performing arts students.

The prevalence of PRMDs in performing arts students, in particular, musicians, is alarmingly high, with estimates ranging from 60% to 90% (1, 4, 6, 18, 32, 33). This vulnerability stems from the repetitive, asymmetrical, and static nature of musical practice, often combined with prolonged hours of rehearsal and limited ergonomic awareness (5, 7, 8, 34–37). Despite these risks, musculoskeletal health education tailored for performing arts students has remained limited, particularly in Eastern European contexts.

Our findings are consistent with earlier research underscoring the importance of early and ongoing musculoskeletal education. For example, the PRESTO program, which integrated behavioral and physical training, led to reduced pain and improved health behaviors in music students (17). Similarly, posture and movement re-education sessions resulted in significant reductions in neck and shoulder pain among conservatory musicians (38). These findings highlight that increased health literacy in posture and spinal mechanics can lead to meaningful physical improvements. The most notable improvement observed in our study occurred in Thomayer's test, a reliable indicator of global spinal flexibility. Participants in the intervention group improved from an average of −7.46 cm (baseline) to −.78 cm (post-intervention), a gain of 6.68 cm, followed by a mild regression to −3.53 cm at follow-up. Parallel improvements were seen in the Schober's test, with values rising from 2.71 cm to 5.60 cm, demonstrating enhanced lumbar spine mobility. Although some regression was noted, the sustained improvement at 24 weeks suggests lasting effects of the intervention, aligning with previous long-term studies on health behavior modification (39, 40). Conversely, the control group exhibited functional decline over the same period. Schober's test results decreased from 3.31 cm to 2.62 cm, and Thomayer's test worsened from −10.12 cm to −10.62 cm. These findings are in line with longitudinal studies that associate the lack of musculoskeletal education with progressive physical deterioration in musicians (11, 41). Thus, the musculoskeletal education program demonstrated both rehabilitative and preventative benefits.

The observed improvements in the experimental group are likely attributable to several interconnected mechanisms. The program's mixed-methods design, which integrates theoretical instruction with hands-on practice, aligns with findings indicating that interactive smart-learning environments can improve posture adherence through real-time feedback (42). This design is further supported by research highlighting the psychosocial benefits of embodied learning, in particular, for individuals experiencing performance-related stress (4). Contributing factors include enhanced proprioceptive sensitivity, greater core stability, and improved neuromuscular coordination, in particular, in dynamic settings, components emphasized in musculoskeletal research (43). The inclusion of breathing-focused interventions, such as diaphragmatic activation, appears to synergize with posture-centered movement routines, facilitating better spinal alignment, and self-correct posture outside of structured training sessions (44–46).

Critical takeaways from our investigation are that few studies have addressed long-term dynamic spine function results following musculoskeletal health education in performing arts students. Despite broad acknowledgment of PRMDs' prevalence (1, 4, 6, 18, 32, 33), affecting up to 90% of musicians, empirical follow-ups beyond the immediate post-intervention phase remain rare (47). The significant regression seen in the control group underscores the essential need for educational inclusion (48). The absence of any structured health education led to a measurable deterioration in musculoskeletal (postural) health. The evidence clearly shows why musculoskeletal health education should be a core part of performing arts training, not just an optional add-on. When arts education is approached in a well-rounded way, it not only improves artistic skills but also boosts academic success and well-being, especially for students in vulnerable situations (49). This is not just about preventing injuries, it is about changing how students think and feel. Music education has powerful cognitive and emotional benefits (50). In the same way, learning about how the body works can help students build both physical strength and stronger sense of control over their own musculoskeletal (postural) health. When students are given practical knowledge and tools they can use, they stop being passive victims of injuries and start taking charge of their well-being (18). Educators, too, benefit from such programs by gaining insight into early markers of dysfunction (51), improving their ability to intervene before injury hampers learning or performance.

Despite the encouraging results of our study, several limitations must be acknowledged. First, the relatively small sample size (*n* = 44, 100%), composed of female music students from 1 institution, restricts the generalizability of the results. Although the homogeneity of the sample helped reduce potential confounding variables, it limits how applicable the findings are to broader groups, such as male students, those from other academic fields, or individuals in different cultural or educational environments (52). Future research should aim to include a more diverse pool of participants to determine whether similar benefits in musculoskeletal function occur across various demographics. Second, while the study featured a follow-up assessment 24 weeks after the intervention, the lack of continued exposure to the musculoskeletal education content during this time likely contributed to the partial regression in measures like Schober's and Thomayer's tests. This points to the importance of reinforcement, as improvements in spine mobility may diminish over time without it. Future work might explore the use of booster sessions or periodic refreshers to support long-term adherence and sustained improvements. Third, the intervention primarily targeted dynamic spine function, yet musculoskeletal health for performing arts students also involves upper limb conditions, neck and shoulder mobility, and ergonomic habits. Therefore, incorporating broader ranges of results, such as pain levels, fatigue, and quality of life (overall), could offer complete understandings of the intervention's benefits. Therefore, future studies should strive for larger and more varied samples, integrate strategies to maintain long-term benefits, broaden the scope of evaluation parameters, and employ stronger experimental methodologies. These improvements would strengthen the evidence base and support the wider integration of musculoskeletal health education into performing arts training programs.

## 5 Conclusions

This study demonstrated that musculoskeletal health education program (structured) can lead to significant short- and long-term improvements in dynamic spine function among female music students in higher education. Participants in the intervention group experienced gains across all spine mobility tests, in particular, in lumbar and thoracic flexibility, with partial retention of these improvements 6 months post-intervention. In contrast, the control group, devoid of any musculoskeletal health education, showed progressive declines in spinal function over the same period. These findings underscore the dual value of musculoskeletal health education as both preventive and rehabilitative strategy in addressing PRMDs, which are alarmingly prevalent among performing arts students. The results also support the integration of mixed-methods approaches, combining theory and practice, to foster sustainable health literacy and musculoskeletal (postural) awareness. The improvements observed are especially relevant given the high physical demands placed on music students, who often lack access to musculoskeletal health education comparable to that provided in sports disciplines. Incorporating musculoskeletal health education into performing arts curricula is not merely advisable, it is essential for fostering both performance excellence and student well-being. The broader implication of this finding lies in its potential to shift the paradigm from reactive treatment of musculoskeletal problems to proactive, education-based prevention. By equipping students with practical strategies for core stability, postural control, and ergonomic awareness, the intervention cultivates habits that extend beyond the academic setting into professional practice. These skills not only help reduce the incidence of performance-related injuries but may also enhance technical precision and physical endurance during demanding performance schedules. Furthermore, the group-based structure and low-resource nature of the program make it easily scalable and adaptable across different institutional contexts. Its long-term effects suggest that even brief, structured educational interventions can lead to sustainable improvements in musculoskeletal function, with implications for curriculum development, health policy, and student support services. As such, integrating similar programs into arts education represents a critical step toward comprehensive health promotion for young performing artists.

## Data Availability

The raw data supporting the conclusions of this article will be made available by the authors, without undue reservation.
